# Cannabidiol: A Brief Review of Its Therapeutic and Pharmacologic Efficacy in the Management of Joint Disease

**DOI:** 10.7759/cureus.7375

**Published:** 2020-03-23

**Authors:** Charles A Gusho, Tannor Court

**Affiliations:** 1 Orthopaedic Surgery, Medical College of Wisconsin Green Bay, De Pere, USA

**Keywords:** osteoarthritis, cannabidiol

## Abstract

Cannabis use in the management of musculoskeletal diseases has gained advocacy since several states have legalized its recreational use. Cannabidiol (CBD), a commercially available, non-neurotropic marijuana constituent, has shown promise in arthritic animal models by attenuating pro-inflammatory immune responses. Additional research has demonstrated the benefit of CBD in decreasing the endogenous pain response in mice subjected to acute arthritic conditions, and further studies have highlighted improved fracture healing following CBD use in murine mid-femoral fractures. However, there is a lack of high-quality, novel research investigating the use of CBD in human musculoskeletal diseases aside from anecdotal accounts and retrospective reviews, perhaps due to legal ramifications limiting the enrollment of patients. The purpose of this review article is to highlight the extent of current research on CBD and its biochemical and pharmacologic efficacy in the treatment of joint disease, as well as the evidence for use of CBD and cannabis in patients undergoing joint arthroplasty. Based on available literature relying on retrospective data and case reports, it is challenging to propose a recommendation for CBD use in perioperative pain management. Additionally, a number of CBD products currently available as supplements with different methods of administration, and it is important to remember that these products are non-pharmaceuticals. However, given the increased social relevance of CBD and cannabis-based medicines, future, prospective controlled studies evaluating their efficacy are needed.

## Introduction and background

With the ever-growing commercial market for cannabidiol (CBD), a derivative of cannabis, there is no doubt that its proposed therapeutic value merits high-quality and novel research, particularly in the management of joint pain. Osteoarthritis is the most common joint disorder in the United States, affecting approximately 27 million Americans [1]. Furthermore, the volume of total joint arthroplasty procedures in the United States has sustained continuous growth over the past two decades, with a projected increase in total hip and knee replacements nearing 71% and 85% over the next 10 years, respectively [2]. Therefore, in conjunction with other, well-studied non-opioid treatment options, CBD may prove to be a beneficial pharmacologic modality for the treatment of joint pain. CBD is a marijuana constituent that has pharmacologic benefits without the additive psychotropic effect of Δ9-tetrahydrocannabinol (THC), another major cannabis ingredient. Currently, anecdotal accounts citing relief of joint pain after smoking cannabis or using CBD exist in the literature, though these data are not corroborated by regulated clinical trials as the legal ramifications may inhibit enrollment in such studies [3]. The following review relays the currently held views on the biochemical efficacy of CBD for the management of inflammation and joint pain and highlights several previous studies that demonstrate a potential human application for CBD in this regard.

## Review

Mechanism of action: cannabidiol

CBD, the major nonpsychoactive component of cannabis, has undergone a bevy of research in murine model organisms, though there is scant, well-vetted evidence of its efficacy in humans. In a study by Malfait et al. in 2000, DBA/1 mice underwent a collagen-induced arthritis (CIA) by immunization with type II collagen (CII) in complete Freund’s adjuvant (CFA) [4]. CBD was then administered after the onset of clinical symptoms, resulting in diminished CII-specific proliferation, IFN-gamma production, and release of tumor necrosis factor. Incidentally, in a separate murine line, the same authors found that CBD was capable of blocking the lipopolysaccharide (LPS)-induced rise in serum tumor necrosis alpha [4]. A subsequent review by Stephen Straus highlighted the aforementioned findings and suggested that CBD is effective when dosed orally or intraperitoneally, noting that it followed a sharp dose-response curve that limits its efficacy range [5]. Thereafter in 2004, Sumariwalla et al. explored the potential antiarthritic effects of a novel, synthetic cannabinoid acid pegged Hebrew University-320 (HU-320). In a prospective manner, these authors immunized DBA/1 mice with bovine CII, injected intraperitoneal HU-320, and assessed the outcomes both clinically and histologically [6]. The results of systemic, daily administration of 1 and 2 mg/kg HU-320 “ameliorated” the established CII-induced arthritis, without any noticeable adverse psychotropic effects [6]. Therefore, these data indicate that cannabinoids such as CBD, in both an anti-inflammatory and immunosuppressive manner, have potent anti-arthritic effects with a subjectively diminished adverse risk profile.

For the literature review, a PubMed Medical Subject Headings (MeSH; MEDLINE) search from 2000 to 2020 was conducted using the following terms: (“Cannabidiol”[MeSH]) and (“Joint Diseases”[Majr]). The search yielded 11 articles, and after reviewing each for accuracy, the focus was narrowed onto eight with the exclusion of those that did not involve CBD. Additionally, Google Scholar was queried using “cannabinoids, joint pain” as key phrases. While the search returned myriad articles from receptor classification to the effects of CBD in animal models, there were no relevant studies regarding any human, clinical data entertaining prospective CBD use and joint pain. In 2006, Blake et al. published an article on the preliminary assessment of the efficacy, tolerability, and safety of a cannabis-based extract called Sativex (GW Pharmaceuticals, Cambridge, UK) used in the treatment of pain from rheumatoid arthritis. Sativex is a cannabis-based pharmaceutical containing THC and CBD, and though a primary limitation of this study was that Sativex was not exclusively composed of CBD, the authors observed a significant analgesic effect with disease suppression following Sativex treatment [7]. In 2011, GW Booz wrote an article on CBD as an emergent therapeutic strategy, attempting to explore exactly how CBD mitigates oxidative stress. His results indicate the endogenous endocannabinoid system acts via CB1 and CB2 G-protein-coupled receptors via lipid ligands, a mechanism that Booz called “ripe for therapeutic exploitation” [8]. Interestingly, the author also notes that CBD has little affinity for the classic endocannabinoid receptor system. In a CB1- and CB2-independent fashion, the actions of CBD on immune cells appear to include the suppression of cell-mediated and humoral immunity. The effect is obtained via blockage of the activation of nicotinamide adenine dinucleotide phosphate (NADPH) oxidase, the delayed wave of reactive oxygen species (ROS) formation, and the associated tumor necrosis factor alpha secretion, and p38 mitogen-activated protein kinases (MAPK) activation [8]. Furthermore, through an unidentified mechanism, CBD was reported to suppress pro-inflammatory signaling and LPS-induced microglial cell migration, while distinctly enhancing other anti-inflammatory pathways [9]. Therefore, given its attenuation of various pro-inflammatory responses in cell models, CBD may certainly have a role in the treatment of pain associated with rheumatoid arthritis via its effects on the immune cell.

Secondly, a more recent exploration into the role of cannabinoids in the treatment of non-rheumatoid arthritis pain suggests that CBD binds to and activates an atypical receptor system entirely. In their article on a novel endogenous receptor called G-protein coupled receptor55 (GPR55), Schuelert and McDougall investigated whether or not the synthetic GPR55 agonist 0-1602, a CBD analog, alters joint nociception in a rat model subjected to acute joint inflammation [10]. The authors induced acute (24-hour) joint pain by injecting male Wistar rats with intra-articular preparations of 2% kaolin and 2% carrageenan. Using extracellular recordings from afferent nociceptive fibers, they found that peripheral administration of 0-1602 reduced the firing of afferent C fibers in response to mechanical rotation of the knee [10]. Though not explicitly translatable to stress-induced osteoarthritic changes in a human knee, this study highlights the role of cannabinoid receptors in joint nociception and suggests a potential relationship between CBD and relief of joint pain in a non-immune fashion. Further evidence for the anti-arthritic role of CBD stems from additional animal studies that evaluate its route of administration and anti-inflammatory effects. Similar to the work produced by Schuelert and McDougall, Hammell et al. investigated a topical CBD application in an attempt to avoid gastric diminution of the drug, hepatic first-pass metabolism, and to achieve greater plasma drug levels outright. The authors describe a favorable transdermal absorption profile when dosed in 0.6-6.2 mg/day, and note that topical CBD significantly reduced joint swelling, limb posture scores, and thickening of the synovial membrane in a dose-dependent manner. Additionally, immunohistochemical analysis of spinal cord and dorsal root ganglia revealed dose-dependent reductions of pro-inflammatory biomarkers, without a concomitant rise in behavior alteration to suggest a psychotropic effect [11]. In light of these data, there emerges a theme. In rodent models, CBD administration has proven anti-inflammatory effects, with a seemingly sharp dose-response peak, no evidence of neurocognitive side effects, and a histologic regression of arthritis in the short term.

Clinical utility: cannabidiol

Currently recommended pharmacologic treatment options for the symptomatic management of osteoarthritis include non-steroidal anti-inflammatories (NSAIDs), low-dose steroids, and viscosupplementation. However, each of these modalities is fraught with side effects when used for long periods of time, and given the insidious time course of osteoarthritis, CBD may prove a useful drug for those with an aversion to other therapies. Additionally, the evidence for viscosupplementation relies on the results of controversial, randomized-controlled trials, and intra-articular preparations have notable contraindications to therapy. Therefore, it is reasonable to suggest that CBD is a safe, useful alternative or adjunct for the treatment of neuropathic joint pain due to secondary osteoarthritis. Osteoarthritis is a progressive disease that results in subchondral bone loss over the years, accelerated by a variety of environmental and genetic factors. In a study by Philpott et al. in 2017, osteoarthritis was induced in male Wistar rats via intra-articular injection of sodium monoiodoacetate (MIA; 3 mg). In addition to its therapeutic effect caused by a decreased joint firing rate and an increased threshold for weight-bearing, the authors demonstrate a prophylactic benefit of 100-300 mcg of CBD as evidenced by a statistically significant reduction of MIA-induced joint pain at a later time point [12]. Despite a small sample size (n = 8), these data are promising and suggest a possible role in prolonging the time course of osteoarthritis, either to the onset of clinical symptoms or to the need for pharmacologic or operative intervention. Therefore, one practical application of cannabinoids including CBD is in the primary prevention of osteoarthritis, or in its preoperative use. In a 2015 study by Kogan et al., CBD enhanced the biochemical properties of healing rat mid-femoral fractures via stimulation of mRNA expression of Plod1 in primary osteoblast cultures, a mechanism well-understood to be involved in collagen cross-linking and bony stabilization [13]. For this reason, along with the evidence presented herein, the orthopedic community has taken interest in CBD, along with other cannabis products, as a potential adjunct for musculoskeletal disease treatment, both in the preoperative and postoperative period.

Clinical utility: cannabis 

Cannabis-based medicines have been employed in the orthopedic practice, though a lack of sufficient data precludes its widespread recommendation. A secondary literature review on cannabis-based therapy in orthopedics was conducted using a PubMed MeSH (MEDLINE) search: (“Cannabis”[Mesh]) and (“Fractures”[MeSH]) OR (“Arthroplasty”[MeSH]). The search yielded nine studies following the exclusion of two that did not meet inclusion criteria or were considered outside the realm of this study (Table 1). Additionally, Google Scholar was queried using the key phrases “cannabinoids, arthroplasty”, which yielded one more recent article by Runner et al. (2020) not found in the initial search.

**Table 1:** MeSH PubMed literature review results: cannabis, fracture, and arthroplasty MeSH: Medical Subject Headings; OA: osteoarthritis; RA: rheumatoid arthritis; TKA: total knee arthroplasty; TSA: total shoulder arthroplasty; THA: total hip arthroplasty; RTKA: revision total knee arthroplasty; CBD: cannabidiol; BMD: bone mineral density; VTE: venous thromboembolism PROs: patient-reported outcomes; BMI; body mass index

Author, year	Design	Aims, methods, and endpoints	Sample size	Importance
Kogan et al., 2015 [13]	N/A	Whether CBD enhances the biomechanical properties of healing rat mid-femoral fractures	N/A	CBD stimulated mRNA expression of Plod1 in primary osteoblast cultures and collagen cross-linking
Richardson et al., 2008 [14]	Cohort	Synovial endocannabinoid expression between healthy and non-healthy (OA and RA) groups	N = 45 total patients; 32 patients with a clinical diagnosis of OA, 13 patients with a clinical diagnosis of RA	Increased CBD1 and CBD2 RNA levels in synovium suggests target for pain and inflammation associated with OA and RA
Best et al., 2015 [15]	Retrospective, National Hospital Discharge Survey	Drug misuse outcomes of primary total hip and knee arthroplasty	N = 13,163 with no drug history; n = 8,366,327 with a drug history	Drug misuse group had higher odds of in-hospital complications
Moon et al., 2019 [16]	Retrospective, National Inpatient Sample, 2010-2014	Marijuana use and in-hospital mortality in commonly billed orthopedic surgeries	N = 9,561,963	Marijuana use was associated with decreased mortality in patients undergoing THA, TKA, TSA, and traumatic femur fixation
Jennings et al., 2019 [17]	Retrospective	Self-reports of use in total joint arthroplasty (500 before and 500 after the legalization in Colorado)	N = 1,000	Legalization of marijuana has led to more users or more patients willing to report its use
Roche et al., 2018 [18]	Retrospective, PearlDiver Medicare database	Effects of drug abuse on revision TKA	N = 2,159,221	Drug abuse patients, including cannabis, are at increased risk for RTKA
Vakharia, et al., 2019 [19]	Retrospective, database retrieval	Whether patients with cannabis use disorder undergoing primary TKA have higher rates of VTE, readmissions; and costs	N = 18,388	Patients with cannabis use disorder have higher rates of VTE complications, readmission rates, and cost
Jennings et al., 2019 [20]	Retrospective	Primary unilateral TKA PROs with minimum 1-year follow-up, who self-reported cannabis use	N = 71	Cannabis use does not influence (adverse or beneficial) short-term outcomes in patients undergoing primary TKA
Sophocleous et al., 2017 [21]	Cross-sectional case control, UK primary care database	Heavy and regular cannabis smokers and BMD scores	N = 56 moderate smokers, N = 144 heavy smokers; matched to 114 cigarette smokers	Heavy cannabis use (>500 lifetime uses) is associated with low BMD, low BMI, high bone turnover, and an increased risk of fracture

CBD acts in a non-endocannabinoid fashion, distinguishing it from THC, the psychoactive ingredient of cannabis. Therefore, cannabis may have a distinct utility profile from CBD. Despite the clinical and preclinical evidence of cannabis-based medicines in combating inflammatory disease, legal ramifications of its use inhibit high-quality, prospective, controlled trials evaluating patient-reported outcomes as a primary endpoint. Retrospective studies have attempted to ascertain the relationship between drug use and postoperative complications following total arthroplasty. In a study by Best et al. in 2015, postoperative total hip and knee patients with a documented history of drug misuse (cocaine, cannabis, amphetamines, and opioids) had greater odds of incurring longer hospital lengths of stay, infection risks, and mortality [14]. Within large database claims such as this, though, cannabis use was likely not the sole culprit for risks of complications, and additional studies have attempted to understand the influence of specific marijuana use on postoperative outcomes in joint arthroplasty. In 2019, Moon et al. conducted a National Inpatient Sample (NIS) database study of 9.5 million inpatients undergoing five common orthopedic procedures: total hip, total knee, and total shoulder arthroplasties, spinal fusion, and traumatic femur fracture fixation. They identified a history of marijuana use disorder in 0.28% of total inpatients from 2010 to 2014, though only within patients undergoing total hip, knee and shoulder arthroplasties, and femur fixation do they describe a decreased odds of inpatient mortality [15]. Needless to say, the association between cannabis use and orthopedic surgical procedures remains unclear. Substance abuse can have a strong negative impact on the outcomes of arthroplasty, though cannabis and CBD both have demonstrated biochemical and therapeutic benefits. Therefore, given its increasing social relevance, prospective, randomized data is needed in this regard.

In orthopedic medicine, the benefit of adjunct CBD and cannabis is likely greatest in an otherwise healthy patient committed to a full, functional recovery, and these data cannot be derived from retrospective database studies. There is no doubt that its recreational use is growing, especially in states where it has been legalized. In a study by Jennings et al. in 2019, 1,000 records of patients undergoing primary total joint arthroplasty (500 consecutive before and 500 consecutive after the legalization of the commercial sale of marijuana in Colorado) were analyzed. The authors describe an increase in self-reported cannabis use from 1% to 11% following its legalization, attributable either to increased use, or increased self-reporting, given the lack of legal ramifications [16]. However, the significance of these results remains unclear. The potential clinical utility of cannabis-based medicines extends from the pre-operative period, following a diagnosis of osteoarthritis, into the peri-operative stage, including postoperative follow-up. No current evidence exists on whether or not cannabis-based medicines including CBD prolong time to total arthroplasty following a diagnosis of osteoarthritis. However, in a Medicare database study by Roche et al. in 2018, patients with a history of drug abuse including cannabis (cannabis use disorder) were at a significantly increased risk for revision total knee arthroplasty than a matched cohort [17]. Furthermore, retrospective studies by Vakharia et al. and Jennings et al. in 2019 note that patients with cannabis use disorder have statistically significant higher rates of venous thromboembolism (VTE) complications and costs, without an increase in postoperative range of motion or a mean improvement in mental and physical scores [18,19]. Similarly, in a prospective cohort of patients undergoing primary, unilateral THA/TKA enrolled in a single institution in California, where THC and CBD are legal, the authors describe a wide variety of usage patterns of THC/CBD; however, they note that between CBD/THC users and non-users, there was no significant difference in the length of narcotic use, narcotic pills consumed, average postoperative pain scores, the percentage of patients requiring a refill of narcotics, or length of stay [20]. In conjunction with sufficient literature that suggests that endocannabinoids have utility in mitigating the anti-inflammatory effects of osteoarthritis, these data highlight the potential pre-operative and preventative use for cannabis-based medicines as opposed to the postoperative utility.

Bone mineral health: endocannabinoids

In an orthopedic practice, joint replacement is quite prevalent, though fracture care is also a potential, high-volume area of interest for the use of cannabis or CBD. Though the current evidence is scant, in the aforementioned animal model study conducted by Kogan et al., THC was noted to potentiate the CBD-stimulated work-to-failure at six weeks post-fracture, followed by attenuation of the CBD effect at eight weeks, which would be a primary indication for use of cannabis in the setting of a fracture. Additionally, in a cross-sectional case-control study by Sophocleous et al. in 2017, heavy cannabis users (>500 lifetime uses) had lower hip and spine bone mineral density, lower BMI, and higher bone turnover, and increased fracture risk than a matched cohort who reported <500 lifetime cannabis uses [21]. It is not clear whether the effects on bone turnover are mediated by the endogenous cannabinoid receptor pathway, nor which amount of cannabis use results in optimal bone health. However, these data collectively suggest a potential application for CBD or light cannabis use in patients who are susceptible to fractures or who pursue fracture management through non-surgical interventions.

Study limitations: the present study

The present study represents a brief literature review using MEDLINE (PubMed) and Google Scholar search engines. This review did not satisfy criteria set forth by the Preferred Reporting Items for Systematic Reviews and Meta-Analyses (PRISMA), and additional databases such as EMBASE and Web of Science were not queried, which may decrease the guarantee of adequate and efficient coverage in the retrieval of articles. A time period from 2000 to 2020 was set forth in the literature review, and studies were excluded if they were not written in English, were duplicated, or lacked relevance to this review. The authors believed that searching MEDLINE and Google Scholar would highlight articles with relevance to CBD and joint pain, though it is possible that human clinical data may be uncovered via other search engines as well. The limitations of the articles within this review are discussed intermittently throughout.

Study limitations: collected articles

While animal-based model studies are important for the classification of endocannabinoids from a biochemical perspective, the preclinical and clinical human data presented herein have several limitations. First, to the knowledge of the authors, there is one study that attempts to prospectively ascertain the effects of CBD on peri-operative arthroplasty. However, in this study by Runner et al., CBD was not standardized among patients, meaning there was a wide variety of reported usage patterns, and the sample size was relatively small (n = 295) [22]. Future studies must prospectively enroll patients with the intent to monitor primary endpoints after standardized CBD doses and how they affect postoperative outcomes. With respect to the collected articles included in the review on cannabis-based medicine and arthroplasty, several limitations arise. six of the nine studies were retrospective in nature. None of the studies enrolled patients to receive CBD or cannabis-based products in a longitudinal manner, either pre-operatively with a diagnosis of osteoarthritis, or postoperatively in addition to their scheduled pain management plan. As mentioned earlier, legal ramifications likely inhibit high-quality prospective studies, and these studies are needed in the future before recommendations on THC/CBD use with arthroplasty can be made.

## Conclusions

Cannabis has gained widespread popularity following the legalization of its recreational use in several states. CBD, a major non-neurotropic marijuana constituent that is also commercially available, has shown promise in mouse model studies by attenuating pro-inflammatory immune responses. Additionally, recent research has demonstrated the efficacy of CBD in decreasing the endogenous pain response in mice subjected to acute arthritic conditions, as well as improved fracture healing via collagen cross-linking in a murine mid-femoral fracture cohort. However, there is a lack of high-quality, novel research investigating the use of CBD in human musculoskeletal diseases aside from anecdotal accounts. This review highlights the extent of the current research on CBD and its biochemical and pharmacologic efficacy in the treatment of joint disease, as well as the current evidence surrounding cannabis-based medicine and orthopedic joint replacement. Currently, there are no approved pharmaceutical products that contain CBD alone for the management of pain. Based on available literature relying on retrospective data and case reports, it is challenging to propose a recommendation for CBD use in perioperative pain management. Additionally, a number of CBD products are currently available as supplements with different methods of administration, and it is important to remember that these products are non-pharmaceuticals. However, given the increased social relevance of CBD and cannabis-based medicines, future, prospective controlled studies evaluating their efficacy are needed.
